# Editorial: Revealing neural plasticity in responding to non-invasive physical therapies *via* fMRI

**DOI:** 10.3389/fneur.2022.1110063

**Published:** 2022-12-16

**Authors:** Yihuai Zou, Jian Kong, Jiliang Fang, Lijun Bai

**Affiliations:** ^1^Department of Neurology, Dongzhimen Hospital Affiliated to Beijing University of Chinese Medicine, Beijing, China; ^2^Department of Psychiatry, Massachusetts General Hospital, Harvard Medical School, Boston, MA, United States; ^3^Lab for Functional Imaging, Department of Radiology, Guang'anmen Hospital China Academy of Chinese Medical Sciences, Beijing, China; ^4^Department of Biomedical Information Engineering, School of Life Science and Technology, Xi'an Jiaotong University, Xi'an, China

**Keywords:** neural plasticity, non-invasive physical therapy, fMRI, acupuncture, transcranial magnetic stimulation, neural pathway, neural network

Neuroplasticity, which refers to the dynamic character of the brain's neural networks, is fundamental in theories of neural rehabilitation that include changes in the structure and function of the brain. It usually takes a long time to get improved during neurological rehabilitation. Thus, non-invasive physical therapy, with its high safety and long-lasting efficacy, could be an important support in the rebuilding of neurological and psychiatric diseases. Blood oxygen level-dependent (BOLD) functional magnetic resonance imaging (fMRI) reflects changes in deoxyhemoglobin concentration resulting from task-induced or spontaneous regulation of neural metabolism. With the development of fMRI, increasing numbers of neuroimaging biomarkers have been tested. This topic contains 15 papers that investigated brain structural and functional changes in neural plasticity associated with non-invasive physical therapies.

## Non-invasive physical therapies in neurological and psychiatric diseases

Transcranial magnetic stimulation (TMS) is a non-invasive treatment technique that uses a magnetic field to activate the brain cortex. An et al. used continuous theta-burst stimulation (cTBS) to induce an inhibitory aftereffect in the motor cortex in short time periods and observed the offline effect of cTBS-induced changes to the left posterior inferior frontal gyrus (pIFG) in healthy subjects. Further research will examine the therapeutic effects of cTBS on the right Broca's homolog area.

Recently, TMS has been used to treat mental disorders such depression, obsessive-compulsive disorder, schizophrenia (SCZ), and other brain-related conditions. Chen et al. conducted a randomized, double-blind, placebo-controlled study of rTMS in patients with stress-related depression. They found that 10-Hz rTMS over the left DLPFC improved the cognitive function of patients with stress-related depression. Ning et al. identified several potential TMS targets on the brain surface (with dlPFC and SMA being the most promising regions) and locations on the scalp for treating SCZ patients.

As a non-invasive peripheral neuromodulation method, non-invasive transcutaneous vagus nerve stimulation (taVNS) has drawn the attention of the investigators. Ma et al. proposed direct stimulation of the afferent nerve fibers on the ear should have a comparable impact on lowering depression symptoms as traditional invasive VNS, but without the requirement for invasive surgery. Meanwhile, He et al. observed a modulational effect of taVNS on the pre-frontal cortex in patients with chronic insomnia.

Continuous positive airway pressure (CPAP) is a conventional non-invasive physical therapy to eliminate snoring and prevent sleep apnea. Liu et al. proposed that short-term CPAP therapy may not be enough, and further longitudinal studies are warranted to explore the reversible alterations in white matter (WM) microstructure in the brain after long-term treatment.

Acupuncture, a key component of traditional Chinese medicine, has become an popular alternative method for stroke rehabilitation and has been used for overall wellness, including pain relief. It can be practiced not only in traditional forms but also in compound methods. Wang et al. conducted traditional acupuncture at acupoint (GB 34) on stroke patients and showed that acupuncture could improve the neurological motor deficit symptoms based on the group-level post-stroke neuroplasticity. Besides, traditional acupuncture was verified by Ning et al. as being able to widely modulate all brain networks and reverse the specific network functional connectivity associated with sleep deprivation. To enhance the intensity of the stimulation during acupuncture, Xu et al. attached a small electrode to the needles as they applied the electroacupuncture (EA) treatment in post-stroke aphasia. The EA procedure involved a continuous, constant-amplitude pulse wave as the stimulation waveform, with 2 mA of electric current and 150 Hz of frequency recorded. Zhan et al. proposed another option for EA parameters in the treatment of Alzheimer's disease (AD), in which acupoints (DU24 and GB12) were electrically stimulated as a pair with a disperse-dense wave of 2/50 Hz and 0.5 mA. They found that treatment with EA and donepezil in Alzheimer's disease patients could primarily increase spontaneous neural activity in specific brain regions, which was associated with improved cognitive function.

Acupuncture paired with exercise training is a type of integrated Chinese and western therapy. Ocular Acupuncture Kinesitherapy (OAKT) is a form of moving acupuncture in which exercise training is carried out while ocular acupuncture needles are embedded in the orbital tissues. Zhang D. et al. introduced a prospective functional neuroimaging and neurotic electrophysiological study with a case-control design in which they assessed the central-peripheral neural function alterations and tried to explain the central-peripheral coupling effect of OAKT on post-stroke dyskinesia. Meanwhile, the role of Tai Chi Chuan, another part of traditional Chinese medicine and often referred to as “meditation in action,” in treating or preventing a range of diseases, is becoming more and more clear. Li et al. found individuals with chronic fatigue syndrome may benefit from practicing Tai Chi Chuan to enhance their quality of life.

## Biomarkers represent neural plasticity as reflected by fMRI

Neural plasticity, which includes structural and functional changes through growth and reorganization, has significant implications for healthy development, learning, memory, and recovery from brain damage. Imaging biomarkers have been applied to estimate structural and functional neural plasticity in an efficient manner.

Functional plasticity refers to the brain's ability to alter and adapt the functional properties of neurons. Activation of the brain regions results in a local increase in oxygen delivery that exceeds the actual metabolic demand. An et al. reported language-related brain regions' activation. Functional connectivity (FC) is defined as the temporal coincidence of spatially distant neurophysiological events since 1994, which was analyzed in most of our reviewed studies. Functional connectivity has opened up another dimension for the analysis of fMRI data. Li et al. conducted Granger causality analysis (GCA) on FC results to show the causal relationship between specific brain networks. Xu et al. adopted independent component analysis (ICA) and general psychophysiological interaction (gPPI) to investigate static and dynamic FC changes both at the level of a region of interest (ROI) and at the large-scale network level. Zhang J. et al. conducted characteristic analysis to classify the prognosis of patients based on the Z values of regions resulting from FC analysis. Further, Ning et al. combined FC and network analysis to elucidate the large-scale brain organization and indicate that this combination might be a potentially powerful approach to understanding brain functional architecture. Wang et al. innovatively introduced intersubject functional correlation (ISFC), a new analytical method for revealing the FC of stimuli in various brain regions across subjects to seek a common activation and suppression pattern triggered by interventions. Such analyses of cross-subject studies may help optimize acupuncture regimens based on the common features of particular patients' conditions. Amplitude of Low Frequency Fluctuations (ALFF) and fractional Amplitude of Low Frequency Fluctuations (f/ALFF) are neuroimaging methods used to find spontaneous fluctuations in the BOLD-fMRI signal intensity for a certain region in the resting brain. The regional homogeneity (ReHo) method was used to estimate regional activation patterns through indices of localized concordance. Since their effectiveness and robustness in analysis, the ALFF, fALFF, and ReHo methods have been used in five articles in this topic.

Structural plasticity is often understood as the brain's ability to change its neuronal connections. The changes in gray matter proportion and white matter fiber form are considered examples of structural neuroplasticity. Sun et al. quantitatively analyzed the cortical thickness (CT) and cortical surface area (SA) of infants with global developmental delay (GDD), draw the developmental trajectory maps, and analyze the hemispheric asymmetry to help find the brain structural changes related to the disease. The commonly used indexes of white matter fiber integrity assessment, including fractional anisotropy (FA), mean diffusion coefficient (MD), axial diffusion coefficient (AD), and radial diffusion coefficient (RD), were evaluated by Liu et al. to feature changes in WM microstructure in patients. Since multimodal neuroimaging methods may provide more information than single imaging tool alone, articles in our topic incorporate magnetic resonance spectrum imaging (MRSI) into resting-state fMRI to determine the chemical composition of specific tissue areas *in vivo* by Chen et al. and arterial spin labeling (ASL) combined with three-dimensional time-of-flight magnetic resonance angiography (3D-TOF MRA) into a DTI study to evaluate the regional cerebral blood flow (CBF) and microstructural changes in white matter by Yang et al..

Taken together, neurological rehabilitations are usually outpatient and need a considerable amount of time to develop brain plasticity. Non-invasive physical may be a promising treatment option for those who need continual intervention. There's still room for improvement to fit the various demands of patients. Regarding the evidence in this topic for both structural and functional biomarkers in regional and network-level analyses, multimodal neural imaging may represent a powerful way to conduct research on neural plasticity in the future.

## Author contributions

YZ, JK, JF, and LB organized and proofread the writing of the editorial. All authors read and approved the final version of the manuscript.

